# Quarter-wise proportion and beta-lactam resistance rate of bovine mastitis associated- *Staphylococcus aureus* among infectious episodes in Ethiopia: Systematic review and meta-Analysis

**DOI:** 10.1016/j.heliyon.2023.e18180

**Published:** 2023-07-17

**Authors:** Melkie Dagnaw Fenta, Firdyawukal Abuhay Tafere, Atsede Solomon Mebratu, Birhan Anagaw Malede

**Affiliations:** a Department of Veterinary Clinical Medicine, College of Veterinary Medicine and Animal Sciences, University of Gondar, Gondar, Ethiopia; b Department of Veterinary Microbiology, College of Veterinary Medicine and Animal Sciences, University of Gondar, Gondar, Ethiopia; c Department of Veterinary Pharmacy, College of Veterinary Medicine and Animal Sciences, University of Gonda, Gondar, Ethiopia; d Department of Veterinary Pathobiology, College of Veterinary Medicine and Animal Sciences, University of Gondar, Gondar, Ethiopia

**Keywords:** Bovine mastitis, Beta-lactam, Pooled prevalence, Resistance, *Staphylococcus aureus*

## Abstract

**Background:**

Bovine mastitis, a condition with multifactorial etiology, imposes a significant economic burden on the dairy sector in Ethiopia, with *Staphylococcus aureus* (*S. aureus*) being one of the leading etiologic agents. The acquisition of a compiled source of information concerning *S. aureus* is imperative in order to enhance the control and prevention strategies, as well as to facilitate the successful implementation of the national action plan aimed at curbing antimicrobial resistance by the year 2025. Thus, the primary objective of this meta-analysis was to comprehensively summarize the estimates of the proportion and beta-lactam resistance profile of *S. aureus* in bovine mastitis in Ethiopia.

**Methods:**

electronic bibliographic data such as PubMed, Web of Science, HINARI, Google Scholar, and other databases were used to search articles and quality assessment was performed using the AMSTAR-2. The pooled proportion, the rate of beta-lactam resistance, and a 95% confidence interval were calculated with a random effects model using *STATA 17* statistical software. Funnel plots, and Eggers were used to assess publication bias.

**Results:**

Twenty-six (26) cross-sectional studies were included in this meta-analysis. The overall pooled proportion of *S.aureus* was 35% (95% CI: 0.31 to 0.41). Considerable heterogeneity was observed in the included studies (*I*^*2*^ = 90.75%; P < 0.01). The subgroup analysis of the study region showed significant differences. The highest estimated regional pooled proportion of bovine mastitis-associated *S.aureus* was 40% in the Amhara and Tigray regions. Funnel plot and Eggers results showed no statistically significant publication bias (Eggers test: p = 0.5656) in estimating the proportion of *S.aureus* infections in association with bovine mastitis. A total of 14 articles were included to estimate beta-lactam antimicrobial resistance. The estimated pooled beta-lactam antimicrobial resistance rate of *S.aureus* was resistance to penicillin at 75%, followed by amoxicillin at 67%, ampicillin at 50% and cephalosporin at 57% were evaluated in the treatment of *S. aureus*. Therefore, the present meta-analysis has revealed that the prevalence of bovine-associated *Staphylococcus aureus* and its resistance to beta-lactam antibiotics are alarmingly high in the region of Ethiopia. This further emphasizes the vital necessity of implementing effective preventive measures to reduce the incidence and spread of this pathogen across the entire nation.

## 1 Introduction

Bovine mastitis is one of the major and serious diseases that has a significant impact on dairy production [1,2]. The issue of bovine mastitis requires special attention for farmers residing in low-income countries, including Ethiopia. Losses due to mastitis include reduced milk production, condemnation of milk due to antibiotic residues, veterinary costs, and culling of cows infected over a long period of time [3]. Bovine mastitis is mainly categorized as subclinical and clinical based on the level of severity.

According to Ref. [4] production losses due to subclinical mastitis in Ethiopia accounts for over 90% of the total milk production loss [5] and are estimated at US$38 per lactation per cow from crossbred dairy cows. In addition, it has public health implications; with serious zoonotic potential due to the excretion of zoonotic pathogens and their toxins in dairy products [6]. Mastitis is caused by a wide range of pathogens including bacteria, fungi and viruses. At least 137 etiological agents that can cause mastitis in large domestic animals have been studied, with bacteria being the main culprits [7]. *Staphylococcus aureus* is one of the most important contagious causative agents of bovine mastitis and is responsible for milk spoilage [8].

Another serious public health impact of *S. aureus* is drug resistance. People with methicillin-resistant *S.aureus* (MRSA) infections have a 64% higher mortality rate than people with drug-sensitive infections. Methicillin resistance is caused by the acquisition of the *mecA* gene. This gene encodes an alternative penicillin-binding protein called *PBP2A*, which has a low affinity for beta-lactam antibiotics [9].

Numerous investigations have documented the frequencies of *Staphylococcus. aureus* resistance across diverse nations. In the United States, a cross-sectional investigation was carried out during the year 2005 at the annual American College of Veterinary Internal Medicine forum. The primary objective of this study was to ascertain the extent of methicillin-resistant *S. aureus* colonization among Veterinary personnel. Results indicated that 7% of veterinarians and 12% of technician attendees were colonized with MRSA [10]. Rates were above 70% in South Korea and Vietnam and below 50% in Portugal, Greece and Italy. In Egypt, a study found a prevalence rate of 17.2%. Another study showed that 70–73% of *S. aureus* strains isolated from various foods were resistant to -lactam antibiotics such as penicillin and ampicillin. A study conducted in South Africa found that the prevalence of methicillin-resistant *Staphylococcus aureus* (MRSA) on commercial farms was 5.7–7%. In other African countries, MRSA prevalence rates were higher in Ethiopia (60.3%), Nigeria (28.57%) and Morocco (15%), while lower prevalence rates were recorded in Kenya (7.8%) [11].

*Staphylococcus aureus* is usually affects different body parts of dairy animals, including the head, skin, and nasal mucosa. However, an infected udder quarter remains the main reservoir of infection for non-infected animals during the milking time through contaminated milkers’ hands (developing countries), and milking machines in the case of developed countries [12].The identification and isolation methods, S.aureus could be differentiating from other species based on the formation of coagulase, the fermentation of mannitol, and trehalose [13]. Coagulase test may have the ability to identify S.aureus in more than 95% of all coagulase-positive staphylococci in bovine mastitis. However, it is not a 100% confirmatory diagnosis of *S. aureus* in mastitis infection. *Staphylococcus.aureus* is the most common contagious and zoonotic bacterial infection causing mastitis which results from high production loss in Ethiopia. Several investigations have been documented about the prevalence of *S. aureus* infection in bovine mastitis in different regions and different period of time, with prevalence at cow, herd, and quarter levels. According to most recent reports, the prevalence of *S.aureus* in clinical and sub-clinical bovine mastitis ranges from 10% to 66.07% [[14], [15], [16]] have been studied. Moreover, some investigations in the previous year reported that the average infected quarter suffers a 30% reduction in milk yield and at the cow level a 15% loss its production.

Milk is a comprehensive and highly nutritious food source for humans. Cattle are the main contributors to national milk production, accounting for 85–89% of total production [17,18]. Nonetheless, in several developing countries, including Ethiopia, the quality of dairy products has become a significant health concern for consumers, particularly infants and children. *S. aureus* is a major pathogen of the disease [19] and also the third most common foodborne pathogen worldwide, posing a significant threat to animal husbandry, and public health [20]. This results in a loss of up to €300 animals per cow per year, leading to an increase in clinical, subclinical and recurrent mastitis in cows. The situation is particularly alarming in underdeveloped countries such as Ethiopia, where there is a high prevalence of infectious diseases, limited surveillance networks, problems with laboratory capacity, and poor diagnostic practices [21]. The most worrying aspect of the situation is that contaminated milk may contain antimicrobial-resistant *S. aureus*, which poses a significant health risk to consumers and is recognized by international health organizations as one of the most critical health issues of the 21st century [22]. The most likely reason for the high prevalence of *S. aureus* is the lack of routine prevention and control measures for foodborne pathogens implemented by farms, milk collectors and dairy processors [23]. The findings of a survey conducted in central Ethiopia indicated that raw milk consumption was reported by 31.8% of individuals of all ages [24]. Given that milk is considered a complete meal for human consumption and also acts as a medium for microbial growth, it is crucial to ensure that proper hygiene standards are met for milk products. Beta-lactams serve as the first treatment option for bovine mastitis-associated *S. aureus* in Ethiopia [25]. However, improper and repeated use of these drugs has contributed to the emergence of resistant bacteria. *Staphylococcus.aureus* responds poorly to antibiotic treatments and can lead to prolonged infection and antimicrobial resistance [26].

Meta-analyses are conducted with the aim of summarizing existing evidence and informing specific decisions as well as it could be extended to incorporate economic considerations in a decision analysis framework [27]. Understanding the prevalence and antimicrobial resistance rate of *S.*aureus associated with bovine mastitis is crucial in enhancing therapeutic interventions and preventive measures. Therefore, this systematic review and meta-analysis aimed to provide an overall estimate of the proportion of bovine mastitis associated-*S.aureu s*and beta-lactam resistance rate in the available literature in Ethiopia.

## 2 Methods

The present review employed the PRISMA (Preferred Reporting Items for Systematic Reviews and Meta-analysis) 20220 checklist and PRISMA 2020 abstract checklist, as prescribed by the established guidelines [28]. The checklist served to ensure the comprehensive incorporation of pertinent data from the chosen articles, based on the underlying protocols (Supplementary file 1&2).

### 2.1 Search strategy

The literature search was conducted from September 10, 2021, to October 5, 2022. A comprehensive search strategy was made to identify included studies. Databases such as PubMed, Web of Science, Google Scholar, HINARI, retrieved articles and other manual methods were used for literature searches. The research question was “What is the proportion of S.aureus infection in clinical and subclinical mastitis among mastitis-causing pathogens in lactating dairy cows in Ethiopia? CoCoPop (Condition, Context, and Population) framework was undertaken to search for relevant articles. The condition was staphylococcus infection (Co), the context was Ethiopia (Co), and the Population was cows (Pop).

The search strategy included Medical Subject Heading (MeSH) terms and a range of important keywords. The MeSH terms incorporated in this step like *staphylococcus aureus*, staphylococcus infection, cross-sectional studies, prevalence, mastitis /mammary gland infection, bovine/lactating cows, and epidemiology. Then, the Boolean operator “AND /OR” were applied during an online search by combining related keywords/phrases. Searching protocols used were (Staphylococcus OR Staphylococcus infection OR *Staphylococcus aureus*) AND (occurrence OR prevalence OR infection rate) AND (cows OR dairy cows) AND (mastitis) AND (Ethiopia). All identified studies were imported to Endnote 20 software to remove duplicates.

### 2.2 Selection criteria

The establishment of inclusion and exclusion criteria based on quality was enacted before the commencement of the review processes. Subsequently, the two authors (F.A and N·B) independently conducted a sorting of all studies selected through the search strategy. The selection criteria utilized for research articles and published reports were centered on the primary objectives of the study, as outlined elsewhere. Consequently, any study to be included in the meta-analysis was required to satisfy the following eligibility criteria: (i) Studies considered only cross-sectional study designs, (ii) Studies provided only a clear quarter-level estimate of the proportion of *S. aureus* infection in both clinical and subclinical mastitis in cattle, (iii) selection was only made by the prevalence of *S. aureus* in cattle and lactating cows (iv) the methods used to detect and diagnose bacterial mastitis include the California Mastitis Test (CMT) and/or clinical examination method, (v) the isolation and identification of *S.aureus* was performed using standard bacteriological techniques (vi) articles written only in English (vii) the study period after 2008 was considered.

**Fig. 1:**
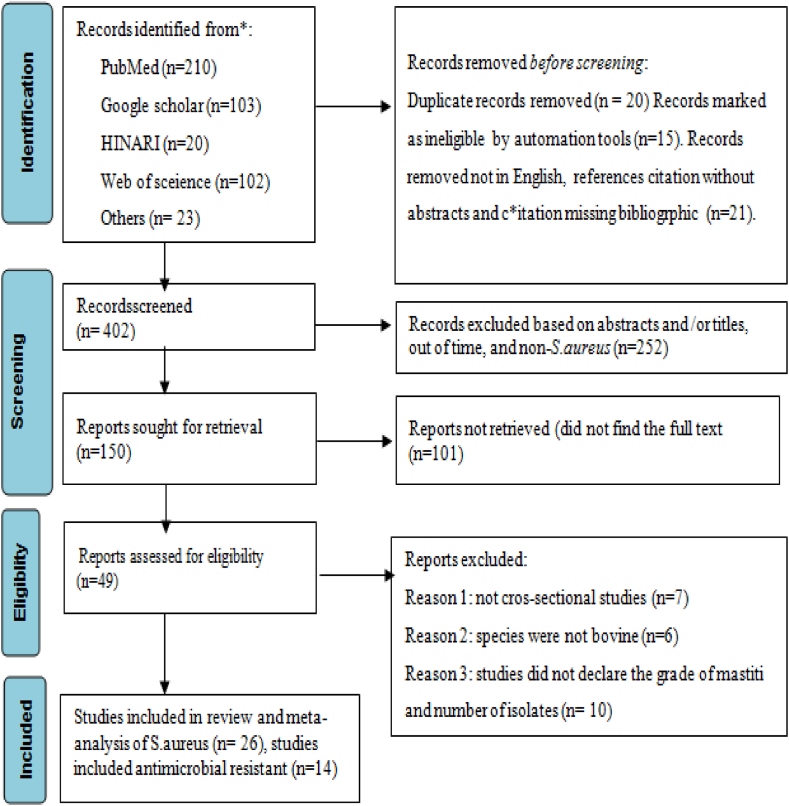
PRISMA flow chart for selection of included and excluded of studies.

Outbreak reports, case series, traditional reviews, cohort and case-control studies, and experimental (clinical) studies, duplication records, the study samples which did not mastitis and the study not involves bacterial identification and isolation, ambiguous sample size, or bacterial isolate quantity not well determined, and the study was conducted out of the defined period (before 2008) were excluded. Finally, the identified inclusion and exclusion criteria were employed for data extraction and meta-analysis, thereby offering a comprehensive study screening strategy and reasons for exclusion (Fig. 1).

### 2.3 Data extraction

From included studies, the following data were recorded by two authors (M.D and B.A) independently: the name of the first author, year of study, study design, study regions, geographical location, and number of cows, number of California mastitis positive quarter, and number of culture positive quarter, and true prevalence of bovine mastitis associated-*S.aureus* infection.

### 2.4 Study quality assessment

As meta-analysis considers the highest level of evidence, quality assessment is performed precisely by standard tools. Quality assessment was done by AMSTAR-2(Supplementary file 3) for this review to check the evidence and methodological quality of the research protocols. The results of meta-analysis rely on the assessment of quality of the included studies in this systematic review and met analysis. Quality assessment was done by two researchers (A.S and F.A) independently.

### 2.5 Data synthesis and statistical analysis

**Fig. 2:**
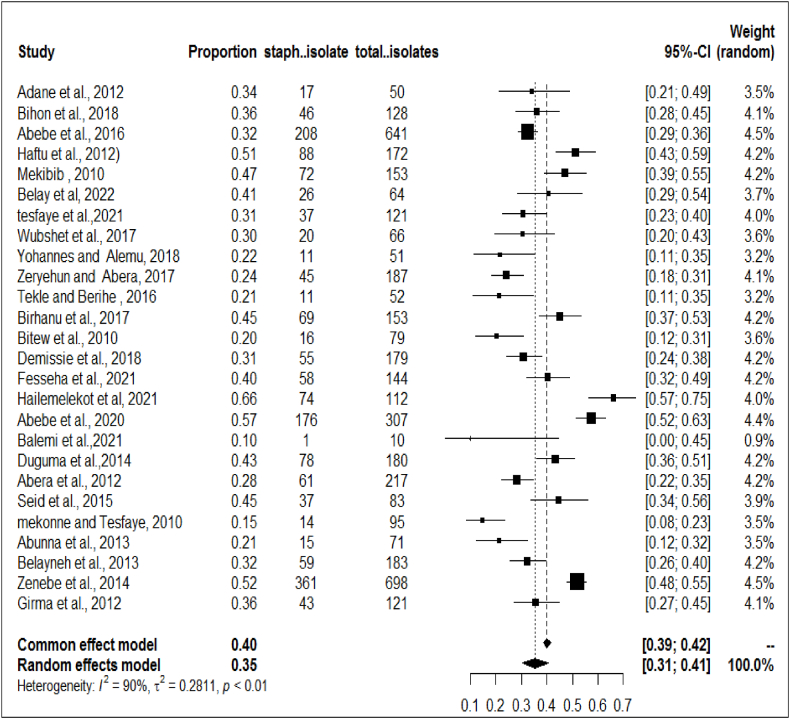
Forest plot for the proportion of bovine mastitis associated *S.aureus* in Ethiopia.

To determine the prevalence of *S.aureus* in clinical and subclinical bovine mastitis in the included studies, the proportion estimates for each study were pooled using a random effects model at a 95% confidence level. A meta-analysis, a subgroup analysis and a meta-regression analysis were performed using STATA-17 statistical software. Between-study heterogeneity was assessed using the Cochran's Q-test (expressed as a p-value) and the inverse index of variance (I^2^), which describes the percentage of the total observed variation between studies that is attributable to heterogeneity rather than chance. The I^2^ values of 25, 50, and 75% show low, medium, and high degrees of heterogeneity, respectively. The presence of heterogeneity between studies was assessed using a forest diagram. The forest plot diagram showed weights, the effect sizes of each study and their CI (Fig. 2). Similarly, subgroup analyzes for the proportion of *S.aureus* in both clinical and subclinical bovine mastitis with study year and study areas were performed to determine specific between-study variability. The academic year category such as (2012), (2013–2017) and (2018) was formed based on the number of articles included.

Furthermore, a meta-regression analysis was conducted to explore the potential factors that may contribute to the heterogeneity among studies, wherein study year, sample size, and regions were employed as covariates. Univariable meta-regression analysis was performed for each selected variable. The sample size was regarded as a continuous variable, whereas the study year and study regions were considered as categorical variables. Publication bias is an inevitable problem in the systematic review and meta-analysis; it is well known as one most important type of biases termed reporting bias, so it is also one of the main threats to the validity of meta-analysis [29]. Small study effects and publication bias were visualized using funnel plot diagrams, Egger's and Begg's test. Egger's regression test is used to test the funnel-plot symmetry, in which a regression model is built, using the standardized estimate of the effect size as a dependent variable and the inverse of the standard error (1/SE) as an independent variable. If the intercept is significantly different from zero, the estimate of the effect is considered biased [30].

## 3 Results

### 3.1 Search results

As viewed in Fig. 1, a total of 458 articles were browsed through different electronic databases and with other methods. A total of 56 articles were removed through duplicate, marked as ineligible and other reasons. A total of 252 articles were excluded through title and abstract screening. One hundred fifty (150) articles reports sought for retrieval and 49 evaluated for eligibility. Finally, only 26 full-text articles for qualitative and quantitative synthesis of bovine mastitis -associated *S.aureus* were included and 14 studies for antimicrobial test. Hence, it has come to our attention that within the selection process of articles, certain articles have been chosen for both the investigation of the prevalence of *S. aureus* and beta lactam antimicrobial resistance. Conversely, other articles were solely employed for either the assessment of *S. aureus* occurrence rate or its antimicrobial resistance.

### 3.2 Characteristics of included studies

This systematic review and meta-analysis was included published reports of staphylococcal infection in bovine mastitis among lactating cows in Ethiopia. It was comprises a total of twenty-six relevant studies for quantitative synthesis of *S.aureus* infection both clinical and subclinical bovine mastitis. All of the included studies were conducted by a cross-sectional study design. The included studies for this systematic review and meta-analysis were conducted between 2008 and 2022 in different parts of Ethiopia. The minimum sample size (quarter level) of individual studies included in this systematic review was 41 [14] while the maximum sample size for the was 1502 [16].

The isolation of *S.aureus* in the included studies were carried out according to the standard microbiological procedures described by NMC(1). All included studies used CMT (sub-clinical mastitis), Bacteriological culture and standard bacterial isolation and identification technique. For this particular study, a comprehensive screening of a total of 8441 cattle was conducted and 10346 quarter-level milk samples analyzed to derive the overall proportion of *S. aureus* infection in Ethiopia. The observed prevalence of *S. aureus* in bovine mastitis ranged from 10% to 66.6%. The detailed characteristics of the studies are well documented in (Table 1).

**Table 1:** Characteristic of the included studies.

Study Name	Year	Region	Location	No. cows	TQE	NQP+	TCP+	Staph+	OBP+	PS*
Abebe et al. [1]	2020	Oromia	Southern	529	2026	729	172	88	84	51
Belay et al. [31]	2020	SNNPRS	Southern	686	1662	132	64	26	38	40
Mekibib [32],	2008	AA	Central	107	428	192	153	72	81	47
Tesfaye et al. [33]	2015	Oromia	Central	384	1536	484	121	37	84	30
Wubshet et al. [34]	2013	Oromia	Southern	28	112	112	66	20	46	30
Zeryehun and Abera [35],	2015	Oromia	Eastern	384	1536	877	187	45	142	24
Tekle and Berihe [36],	2011	SNNPRS	Southern	384	384	70	52	11	41	21
Birhanu et al. [37],	2016	AA	Central	262	1048	170	153	69	84	45
Bitew et al. [38]	2010	Amhara	Northern	302	1208	134	79	16	63	20
Demissie et al. [39]	2015	Tigray	Northern	360	1440	229	179	55	124	30
Fesseha et al. [40]	2019	Oromia	Southern	384	1536	536	144	58	86	40
Hailemelekot et al. [41],	2018	Amhara	Northern	302	1208	126	112	74	38	.66
Abebe et al. [16]	2018	Oromia	Southern	686	2633	773	307	176	131	57
Balemi et al. [14]	2018	Oromia	Southern	60	240	41	10	1	9	10
Haftu et al. [42]	2009	Tigray	Northern	305	1220	187	128	46	82	35
Duguma et al. [43]	2009	AA	Central	90	340	275	180	78	102	43
Abera et al. [15]	2008	Oromia	Southern	245	960	288	217	61	156	28
Seid et al. [44]	2014	Oromia	Southern	358	1422	490	83	37	46	44
Mekonne and Tesfaye [45],	2008	Oromia	Sothern	206	824	790	95	14	81	14
Abunna et al. [46]	2008	AA	Central	331	1324	146	71	15	56	21
Belayneh et al. [47]	2008	SNNPRS	Northern	303	1172	244	183	59	124	32
Zenebe et al. [48]	2011	Tigray	Northern	322	1288	696	698	361	337	52
Girma et al. [49]	2010	Oromia	Eastern	384	1536	1502	121	43	78	35
Adane et al. [50]	2010	oromia	Southern	460	1840	712	641	208	433	32
Bihon et al. [51]	2017	Amhara	Northern	334	1054	238	50	17	33	34

***Note***: G. Location = Geographical Location; St. Design = study design; TQE = Total quarter Examined; NQP^+^ = number of quarter positive: TCP+ = Total culture positive; Staph^+^ = *staphylococcus aureus*, Positive: OBP^+^ = other bacterial pathogen positive; PS* = prevalence of *staphylococcus aureus*.

### 3.3 Meta-analysis and bias assessment

A total of 26 studies were included in meta-analysis and estimated the prevalence of *S.aureus* infection in both clinical and subclinical mastitis. Substantial heterogeneity was observed across the included studies on bovine mastitis associated *S.aureus* infection among lactating cows (*I*^2^ = 90.75%; τ^2^ = 0.2811, *P* < 0.01) and pooled effect size was 35% (95% CI: 0.31–0.41) as shown on the forest plot (Fig. 2). This observation signifies the presence of considerable variations across the investigations. Additionally, subsequent meta-regression and subgroup analysis were conducted to examine the potential factors that may account for the observed variance.

**Fig. 3:**
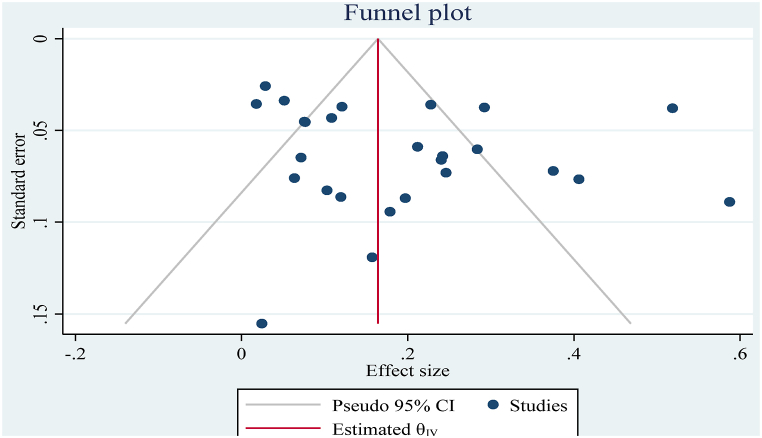
Funnel plot with pooled proportion of bovine mastitis associated *S. auerus*.

The result of the funnel plot (Fig. 3) showed that there was no asymmetrical distribution of studies and almost all studies are under 95% confidence interval which means smaller studies do not tend to be missed.

### 3.4 Subgroup analysis

**Fig. 4:**
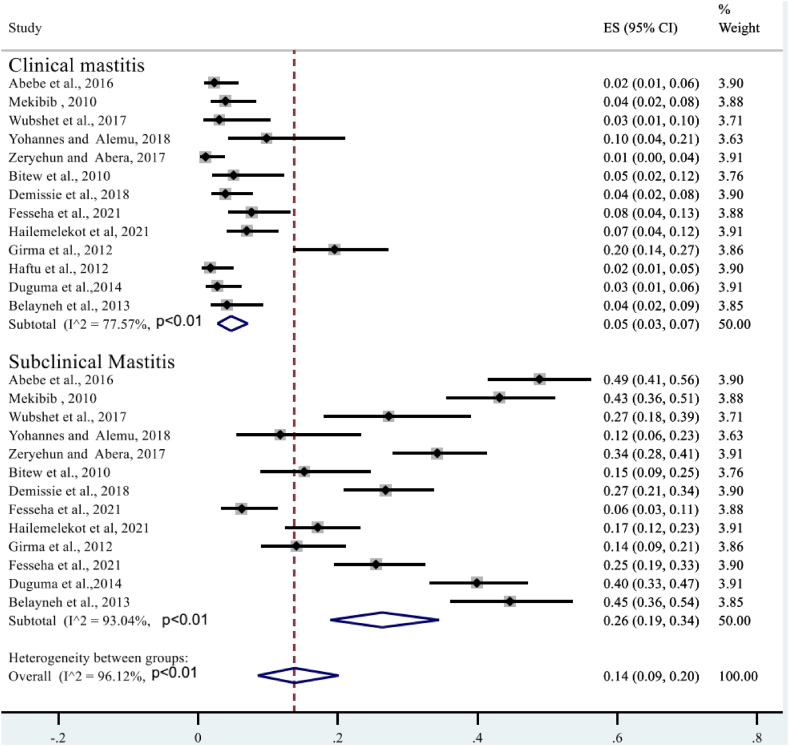
Forest plot and subgroup analysis by degree of mastitis due to *S.aureus*.

**Fig. 5:**
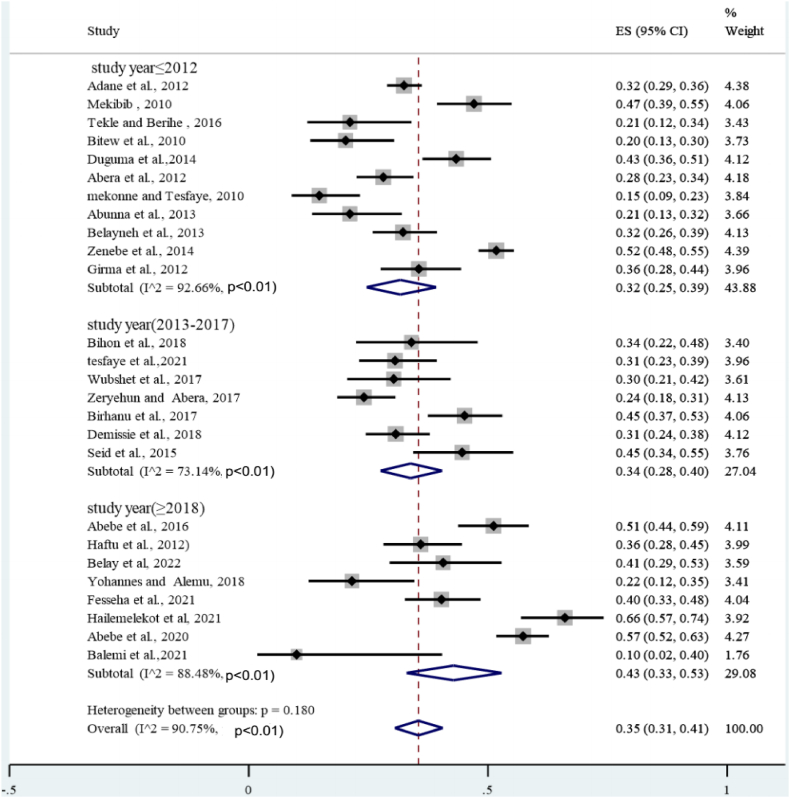
Subgroup analysis of the prevalence of *S. aureus* based on the study year.

**Fig. 6:**
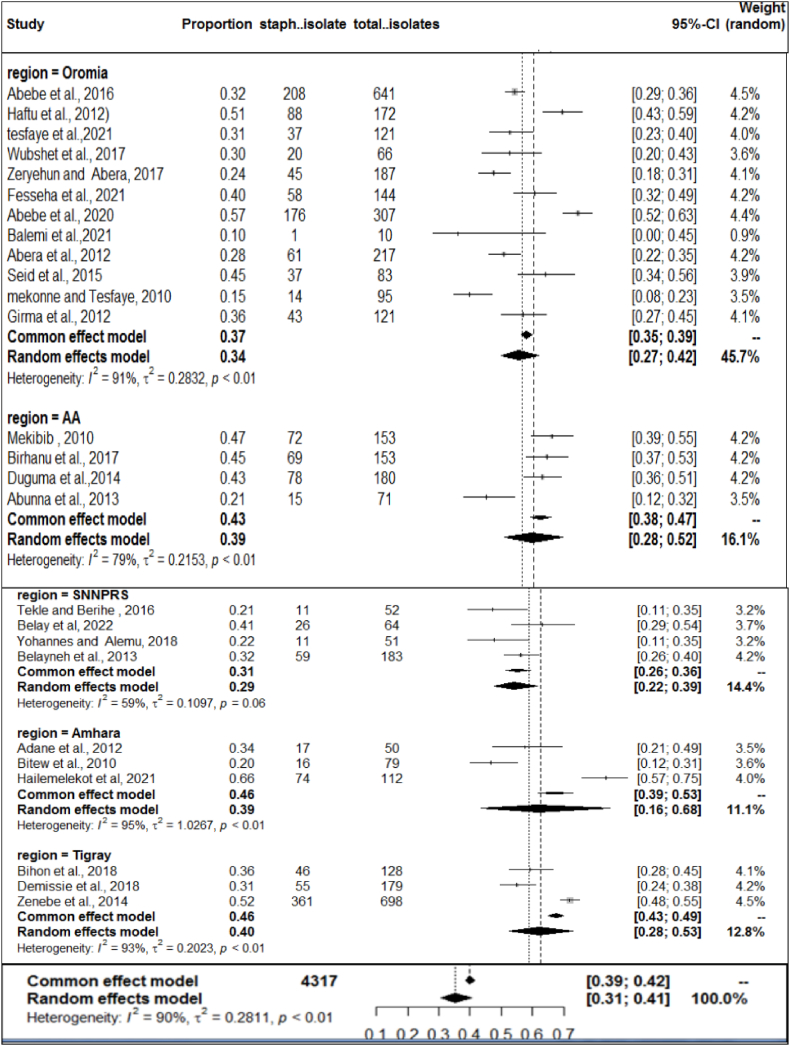
Subgroup analysis of the prevalence of *S.aureus* based on the study region.

**Fig. 7:**
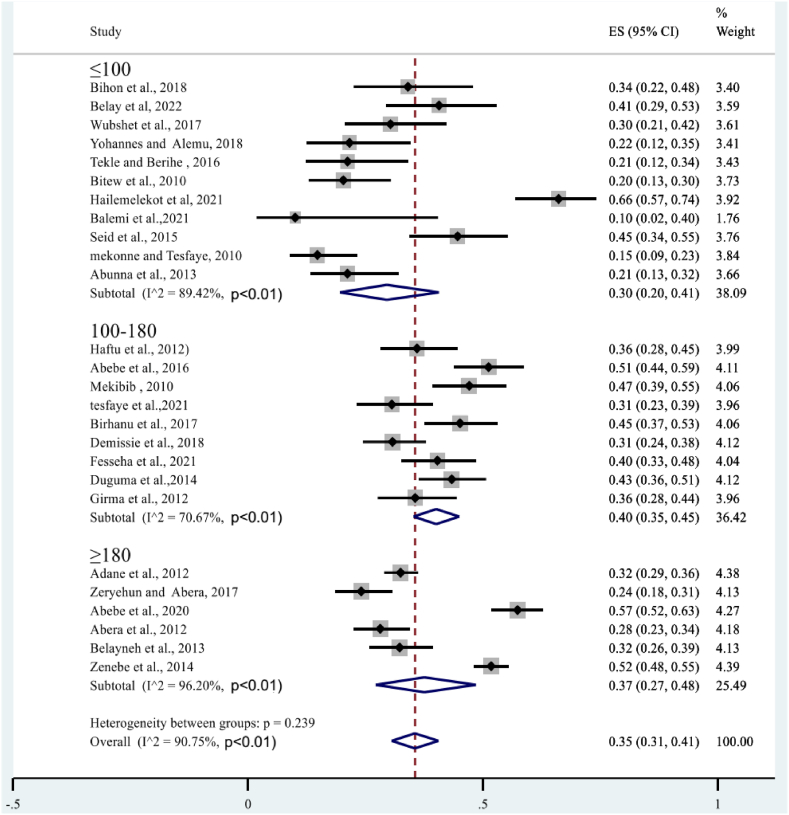
Subgroup analysis of the proportion of bovine mastitis associated *S.aureus* by sample size.

Given the high level of heterogeneity, a subgroup analysis was performed based on mastitis level (clinical and subclinical), year of study, study area (region), and sample size. A sub-analysis of subclinical mastitis studies revealed high heterogeneity (*I*^*2*^ = 96.12%, P < 0.01), whereas clinical mastitis studies showed moderate heterogeneity (*I*^*2*^ = 77.57%; P < 0.01) (Fig. 4). The subtotal percentage of staphylococcal infection was higher for subclinical mastitis (26%) than for clinical mastitis (5%). Sub-analysis also performed by year of study. First, all study years were divided into three groups: (2012), (2013–2017), and (2018), indicating statistically significant heterogeneity in each group. As shown in Fig. 5, the highest heterogeneity (92.66%: P < 0.01) was found in 2012. However, the sub-total proportion of *S. aureus* infection was the lowest at 32% (95% CI: 25–39%). A sub-analysis of the proportion of bovine mastitis associated with *S. aureus* infection by region had revealed the highest heterogeneity (*I*^*2*^ = 91.36%; P < 0.01: Fig. 6) in Oromia regional state. However, subtotal prevalence was the second lowest at 34% (95% CI: 27–42%), followed by SNNPR (29%). Also as exhibited in Fig. 7, subgroup analysis by sample size also showed significant heterogeneity between studies.

**Fig. 8:**
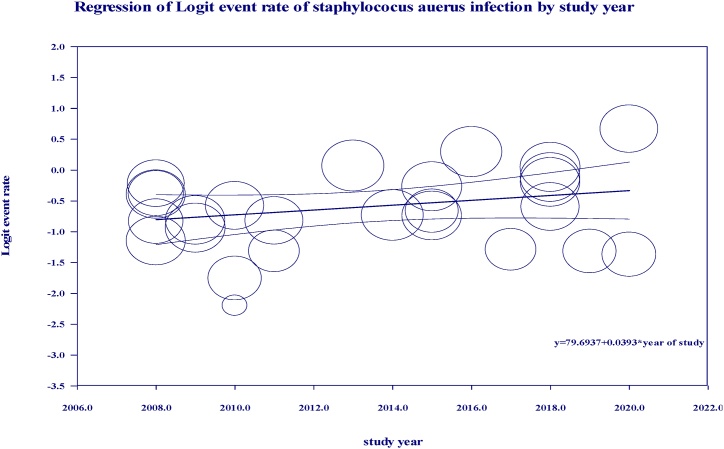
Regression of logit event rate by year of study.

**Fig. 9:**
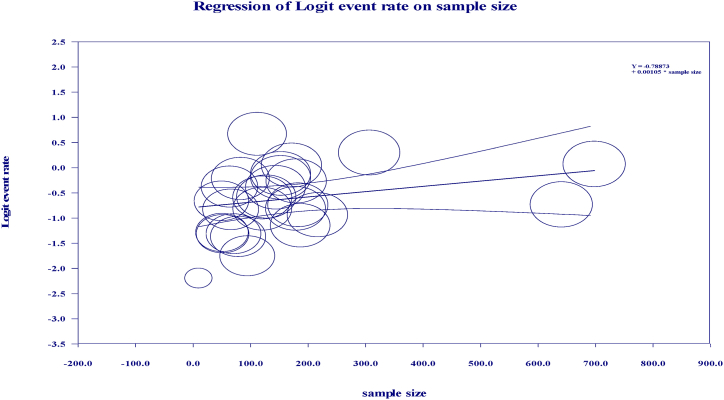
Meta regression plot of sample size versus effect size of the rate of *S.aureus*.

The present study outlines the outcomes of univariable meta-regression analyses in terms of coefficients, p values and Cochran's Q statistics. It is noteworthy that both region and study year exhibited a univariable p value < 0.1. Furthermore, a meta-regression analysis rate was conducted to determine the prevalence of bovine mastitis with respect to study year, which revealed a significant increase in the incidence of *S.aureus* infection. The univariable meta-regression analysis was followed by a multi-variable meta-regression analysis, wherein variables with a significance level of less than 0.1 were selected. The final results of this comprehensive analysis are presented in Table 2. Additionally, Fig. 8, Fig. 9 illustrate the event rate plot on the sample size and year of study, respectively.

**Table 2:** Summary of statistics in multivariable meta-regression.

Variables	category	Coefficient	P -value	95%CI	Q
Study year	≤ 2012	Ref.			247.33
	2013–2017	−0.27	0.00614	−1.421–0.863	
	≥ 2018	−0.118	0.0261	−1.520–1.281	
Region	Oromia	Ref.			42.33
	Amhara	0.30	0.481	−0.593–1.211	
	Tigray	0.62	0.211	−0.386–1.636	
	AA	0.69	0.52	−0.265–1.647	
	SNNPR	0.56	0.32	−0.321–1.321	
Sample size	<100	Ref.			270.41
	100–180	0.76	0.00	0.35–0.45	
	>180	0.82	0.003	0.27–0.48

## 4 Beta lactam antimicrobial resistance rate of *S.aureus*

Antimicrobials are the only option for treating bovine mastitis in Ethiopia. In particular, the main choices of antimicrobials are beta-lactams such as penicillin, ampicillin, amoxicillin, ceftriaxone and cefotaxime. It has been reported that the proportion of glands undergoing bacteriological healing after antimicrobial therapy for clinical mastitis varies widely [52].

Recovery rate is affected by factors related to the individual cow, factors related to management and bacterial associated factors (strain and presence of antimicrobial resistance; also determine the cure proportion [[53], [54], [55]]. The presence of differently sized plasmids was associated with the carriage of multiple antimicrobial resistances. In *S. auerus*, two main classes of plasmids were identified, which contribute to the resistance against antimicrobials and/or virulence. Antimicrobial resistance can be assessing in different methods including agar disc-diffusion assays broth dilution testing and the detection of genes encoding resistance [56].

In the current review we have include a total of 14 studies (Table 3) for beta-lactam antimicrobial (Penicillin, amoxicillin, ampicillin and cephalosporin's resistance) resistance rate of bovine mastitis associated *S.aureus*.

**Table 3:** The various beta-lactam resistance for bovine mastits associated-S.aureus (n = 14).

Author	Antimicrobials	SA + isolated	Resistance	Susceptible	Proportion
Ayana et al. [21]	Penicillin	110	110	0	1.000
	Amoxicillin	110	65	45	0.590
	Ampicillin	110	57	53	0.518
Tassew et al. [57]	Penicillin	53	0	53	0.000
	Cefoxitin	53	28	25	0.472
	Amoxicillin	53	53	0	1.000
Gebremedhin et al. [23]	Ampicillin	40	38	2	0.950
	Amoxicillin	40	38	2	0.950
	Cefotaxime	40	32	8	0.800
Reta et al. [58]	Penicillin	29	28	1	0.034
	Amoxicillin	29	10	19	0.345
Tesfaye et al. [33]	Amoxicillin	12	9	3	0.750
	Ceftriaxzone	12	7	5	0.583
Mekonen and Tesfaye [45],	Penicillin	17	14	3	0.824
	Ampicillin	17	3	14	0.176
Getahun [59],	Pencillin	85	45	40	0.530
	Ampicillin	85	38	47	0.450
Sori [60],	Pencillin	86	75	11	0.872
	Amoxillin	86	40	46	0.460
Moges et al. [61]	Penicillin	27	22	5	0.815
	Amoxicillin	27	22	5	0.800
Haftu [40],	Penicillin	46	38	8	0.830
	Ampicillin	46	14	3	0.304
Belayneh (a) [47],	Penicillin	59	38	21	0.650
	amoxicillin	59	37	22	0.620
Belayneh(b) [62],	Penicillin	32	24	8	0.750
	amoxicillin	32	24	8	0.750
Elemo et al. [63],	Ampicillin	112	62	50	0.550
	Amoxicillin	112	59	53	0.530
	Cefoxitin	112	65	47	0.580
	Penicillin	112	97	15	0.870
overvliet et al. [53]	Penicillin	32	32	0	1.000
	Cefoxitin	32	10	22	0.300

### 4.1 Selection criteria

Out of the total 1907 isolates of *S. auerus*, 1234 were found to exhibit antimicrobial resistance. One article was used multiple times for quantitative data analysis. The selection criteria for the chosen studies were as follows, (1) almost all of the included studies provided a comprehensive overview of the pathogen isolation rates for both clinical and sub-clinical bovine mastitis, (2) each article had conducted multiple antimicrobial susceptibility tests. (3), the chosen articles had isolated *S.aureus* at least once, (4) in each study, at least one beta-lactam antimicrobial was present, (5) the chosen article may or may not be included in the previous section of our meta-analysis which focused on the prevalence of *S. auerus*, (6) we clearly estimated the proportion of antimicrobial resistance and susceptibility, (7) we only selected articles that exclusively used milk as the primary sample for dairy cattle in Ethiopia. Finally, we only included studies that conducted the antimicrobial susceptibility test according to the criteria of the clinical laboratory standards institute.

Articles were deemed ineligible if they met any of the following criteria: (1) duplication; (2) deviation from the topic at hand and a small sample size of less than 60; (3) lack of bacterial identification in the study; (4) presence of non-mastitis diseases in the study samples; (5) an unclear sample size or bacterial isolate quantity in the study; and (6) absence of beta lactam antimicrobials in the study.

### 4.2 Meta-analysis

**Fig. 10:**
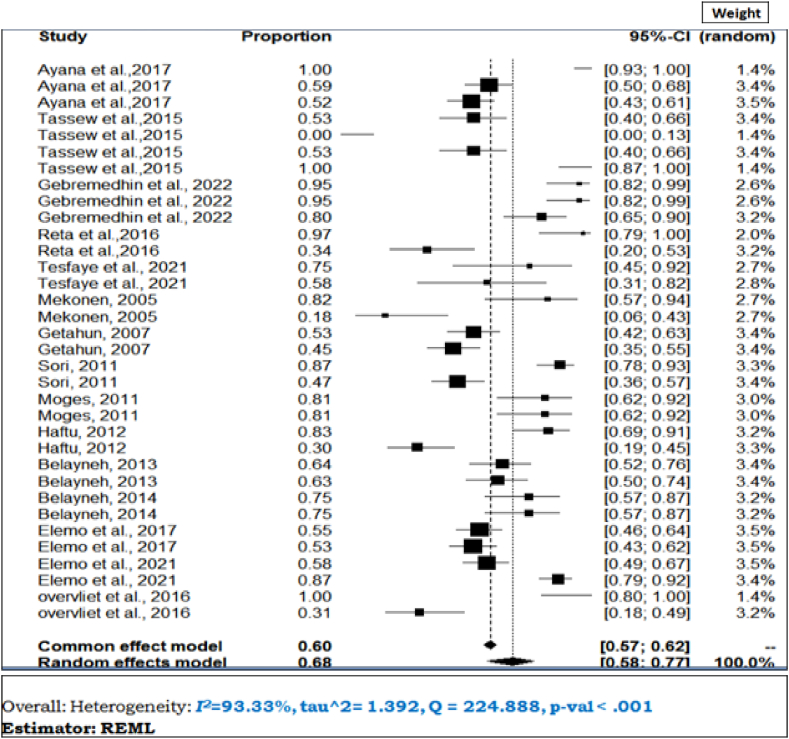
The forest plot, heterogeneity and resistance rate of beta lactamase of *S. aureus*.

Due to expectation of heterogeneity across studies, a random-effects meta-analysis was selected using the total number of S. auerus isolated as total sample size and number of resistance (as event rate). The result of the quantitative analysis (meta-analysis) of 14 studies showed that the individual study resistance rate of S.aureus ranged from 0% to 100%, with the overall pooled prevalence being 68% (95% CI; 58–77%). As shown in Fig. 10, the heterogeneity between and within studies was high (I^2^ = 93.33%; τ^2^ = 1.39, Q = 224.88), P < 0.001).

### 4.3 Publication bias assessment

**Fig. 11:**
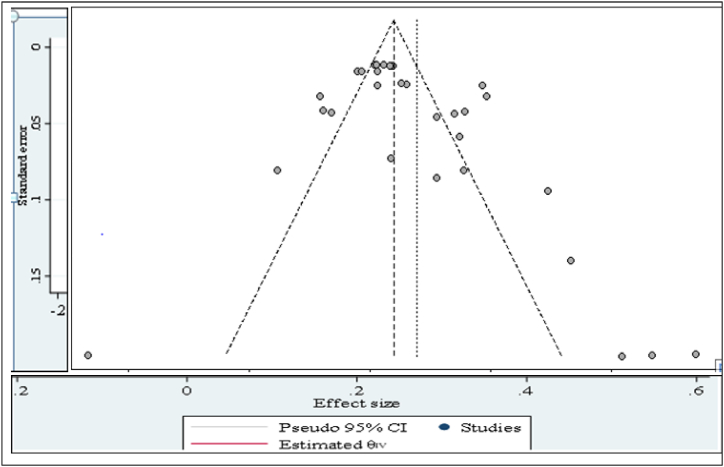
Publication bias assessment of overall beta lactam antimicrobial resistance rate of mastitis associated *S.aureus*.

The funnel plot in Fig. 11 revealed that no asymmetrical distribution of studies and most of the studies are under a 95% confidence interval, the regression test was computed by using-mixed effect Meta regression model and standard error of the effect size as the predictor. Egger's regression test and rank correlation test of the funnel plot asymmetry were confirmed no significant publication bias (b = 39.32; *P* = 0.62) and rank correlation test (Kendall's tau = −0.1277; *P* = 0.2920), respectively.

***Penicillin resistance rate:*** of 14 studies, 12 studies were pooled to estimate the proportion of *S. auerus* resistance to penicillin. In Supplementary F. 1, the overall proportion of *S. auerus* resistance for penicillin was 75% (95% CL; 59–91%) in the treatment of bovine mastitis.

**Heterogeneity:** based on the twelve identified studies, a meta-analysis was conducted to investigate heterogeneity within and across studies using a random effect model. Studies reporting the resistance rate of *S.aureus* penicillin, there was evidence of between-study heterogeneity (*I*^*2*^ = 77.41%, *P* < 0.01).

***Assessment of bias*:** The funnel plot revealed that there was no asymmetrical distribution of articles (Supplementary F. 2), and the majority of studies are within the Pseudo 95% confidence interval. The results of the egger's regression test for small-study effects showed no statistically significant publication bias (Egger's test: b = 0.63, *P* = 0.7653).

***Amoxicillin resistance rate:*** of 14 studies, 10 studies were selected for estimation of the proportion of resistance of *S.aureus* to amoxicillin for the treatment bovine mastitis.

***Heterogeneity***: based on the ten identified studies, a meta-analysis was conducted to investigate heterogeneity within and across studies by using a random effect model. For studies reporting resistance of *S.aureus* to amoxicillin, there was evidence of between-study heterogeneity (*I*^*2*^ = 55.46%, p = 0.02; Supplementary F. 3); the pooled effect estimate was 67% (95%CI: 54–80%) in random effect meta-analysis.

***Assessment of bias*:** the funnel plot was used to assess publication bias, which was confirmed by the Egger regression test (b = 1.37; P = 0.3364) (Supplementary F. 4).

**Ampicillin resistance rate***:* six studies were selected for estimation of the proportion of *S. auerus* resistance to ampicillin in the treatment of bovine mastitis among dairy cows. The overall proportion of *S. auerus* resistance for *ampicillin* was 50% (95% CI; 29–71%).

***Heterogeneity***: according to six identified studies, a meta-analysis was carrying out to examine variability within and across studies using a random effect model. For studies reporting resistance of *S. aureus* in the treatment of ampicillin, there was high variability (*I*^*2*^ = 96.17%, Q = 153; P < 0.01; Supplementary F. 5).

**Assessment of publication bias**: the funnel plot was used to assess publication bias, which was confirmed by the Egger regression test. There was no publication biased because the regression-based Egger test for small-study effects was not statistically significant (b = 0.37; *P* = 0.2364) by using a random effect model with the method of restricted maximum likelihood (Supplementary F. 6).

***Cephalosporin's resistance rate****:* five studies were selected for estimation of the proportion of resistance of *S. auerus cephalosporin* sin treatment of bovine mastitis among dairy cows. As supplementary F. 7 showed, the overall proportion of *S. auerus* resistance rate to cephalosporin was 57% (95% CI; 45–69%) in the treatment of bovine mastitis.

***Heterogeneity***: based on the five identified studies, a meta-analysis was conducted to investigate heterogeneity within and across studies using a random effect model. For studies reporting resistance of *S. auerus* to cephalosporin*,* there was no evidence of between-study heterogeneity (*I*^*2*^=0.00%, Q= 4.40; *P* = 0.35).

***Publication bias***: there was publication biased (Supplementary F. 8) because the funnel plot showed asymmetrical distribution of studies, as well as the regression-based egger test for small-study effects, was also statistically significant (b= -12.32; *P*=0.001). The findings of the various antimicrobial agents, the collective resistance, and the potential for publication bias across all Meta –analysis have been consolidated into Table 4.

**Table 4:** The summary of the beta lactams with pooled estimates, heterogeneity and bias assent.

Type of antimicrobial	Pooled resistance rate	Heterogeneity% (I^2^)	95%CI	Bias(Egger's test)
Penicillin	75	77.41	59–91	b = 0.63, *P* = 0.7653
Amoxicillin	67	55.46	54–80	b = 1.37; P = 0.3364
Ampicillin	50	96.17	29–71	b = 0.37; *P* = 0.2364
Cephalosporin	57	0.00	45–69	b = −12.32; *P* = 0.001

## 5 Discussion

A systematic review and meta-analysis was performed to estimate the pooled proportion of *S.aureus* infection in clinical and subclinical bovine mastitis in lactating dairy cows in Ethiopia. The current study, involving 26 studies, found that the pooled proportion of S. aureus-associated bovine mastitis in Ethiopia was 35% of the bacterial pathogens causing mastitis in lactating cows. Comparable results were reported by Girma et al. [49] reported in Tigray Regional State, Ethiopia.

In view of its global importance, *S. aureus* is widely recognized as a major cause of intramammary infections in dairy cows. This particular study found that the pooled prevalence of *S. aureus*-associated bovine mastitis (35%) found to be almost identical to that which was stated in a meta-analysis conducted in China (36.23%) by Wang et al. [64], figures to be higher in Canada at 20–22% [65] and in the United States at 20.8–23.3% [66] and at 15.7% in Kenya [67]. In contrast, the result was lower than the results reported by Elhaig and Selim [68] in Nigeria at 56.7% and in Egypt at 38.3%. These differences may be related to cow cleanliness, availability of vaccination, farm size, farm management systems, breed of cows and housing styles in different countries and regions are some of the factors contributing to the complexity of mammary health [69].

In terms of study year, the sub-total prevalence of *S.aureus* infection in clinical and subclinical mastitis in each study year category were 32% (>2012), 34% (2013–2017) and 43% (>2018). According to this systematic and meta-analysis the pattern of rate of *S. auerus* infection in bovine mastitis had increased in the subsequent stratification of included studies based on study year. This may due to increased antimicrobial resistance by various mechanisms and it is believed that about 50% of mastitis causing *S.aureus* produces β-lactamase [70].This may related with the transfer of resistant strain among environment, livestock and human. This may also due to with limited therapeutic treatment in Ethiopia and improper use of antimicrobials in veterinary practice. Another justification *S. aureus* biofilm formation is related with diminished antibiotic sensitivity in bovine mastitis, which is attributed to impaired antibiotic penetration through the biofilm matrix and decreased metabolic activity of bacteria within biofilms [71]. Furthermore, several circumstances, such as sub-inhibitory concentrations of various antibiotics [72].And milk components such as lactose and proteases, stimulate *S. aureus* biofilm formation inside the bovine mammary gland [73].

Regarding the study regions, the pooled prevalence of *S. aureus* infection was highest in Addis Ababa 40% whereas lowest in SNNPRS region 29%. The observed variation can be attributed to differences in management practices. Such discrepancies are particularly notable given the infectious nature of *S.aureus*, which is easily transmitted between cows. This is favored by unhygienic milking processes, such as the use of contaminated milking equipment and hands, and the involvement of flies and cross suckles [74].

Regarding level of mastitis, this systematic and meta-analysis indicated that the level of occurrence of *S. aureus* infection was higher (26%) in subclinical mastitis than clinical bovine mastitis 5%. Thus, this result confirmed that *S. aureus* infection has the great economic impact on dairy sector in Ethiopia because subclinical mastitis is the major bottle neck of dairy sector in developing countries [75] *S.aureus* is a very well opportunistic infection that is frequently related to subclinical mastitis and causes significant economic losses due to reduced milk quality and production [76]. Intrammary infection of lactating cows by CC97 strains has been shown to result in asymptomatic, sub-clinical, or persistent infections, increasing the difficulty of pathogen control in dairy herds [77].Although the overall rate of resistance varies widely among antimicrobial agents, resistance of S.aureus to antimicrobial agents is a serious problem. According to this meta-analysis, the overall rate of beta-lactam resistance in Ethiopia was very high (68%). This finding is corroborated by research carried out in Iran [78] and Brazil [79], which demonstrates that *S.aureus* displays a pronounced resistance to β-lactam antibiotics in comparison to alternative antibiotics. This may be because *S.aureus* produces beta-lactamase, which breaks down the drug's beta-lactam ring or it could be the synthesis of a redesigned PBP that has a decreased affinity for the majority of beta-lactam antimicrobials. *Staphylococcus aureus* may have acquired resistance to β-lactam antibiotics through a variety of mechanisms. These include modifications and expressions of penicillin-binding proteins (PBP), β-lactamase synthesis leading to drug inactivation, biofilm formation limiting drug uptake, and expression of efflux pumps reducing drug uptake. Specifically, the production of beta-lactamase by *S.aureus* can catalyze the breakdown of the drug's beta-lactam ring. Alternatively, a redesigned PBP with decreased affinity for beta-lactam antimicrobials may have been synthesized. In cases of bovine mastitis, biofilm formation by *S. aureus* has been linked to decreased susceptibility to antibiotics. This is likely due to reduced antibiotic diffusion through the biofilm matrix and decreased metabolic activity of bacteria within biofilms as suggested by Song et al. [80]. Furthermore, it has not been previously recognized that *S.aureus* could act as an intracellular pathogen. It was not previously known that *S. aureus* could act as an intracellular pathogen. However, there is now substantial evidence to suggest that the bacteria can survive and persist within a variety of mammalian cells, rendering antibiotic treatments ineffective [81]. This mechanism of survival confers a protective advantage to *S. aureus* as it enables the bacteria to evade both the host immune response and antibiotics, thereby rendering the bacteria inaccessible [82].

The prevalence of *S.aureus* resistance to penicillin is widespread in dairy herds across the globe. In the present review, it was observed that penicillin exhibited the highest resistance to *S.aureus* (75%) as compared to other beta lactam. This percentage value was higher than the values reported in Argentina (47.6%), Brazil (69.9%), Turkey (62%), Estonia (61%), France (30%), Hungary (30%), Germany (17%), the United States (10%), and Norway (6%) as reported by Gianneechini et al. [83], André et al. [84], Turutoglu et al. [85], Kalmus et al. [86], Sakwinska et al. [87], Peles et al. [88], Jørgensen et al. [89], Anderson et al. [90], and Tenhagen et al. [91], respectively. However, the resistance percentage was lower than the values reported in Mukaturi and Sululta (35%), Tigray (over 90%), Addis Ababa (95.3%), Hawassa (100%), Iran (100%), and China (26%). This variation may due the importation and distribution of poor quality drugs to the country [92]. This is also related to the fact that β-lactams rank first regarding the number of compounds commercially accessible and in terms of their use in the treatment of bovine mammary infections. In addition, penicillin belongs to the β-lactam category of drugs; the drug has been adopted for long-term and repeated administration in cattle, for example, for the treatment of diarrhea and other diseases, which may bring in increased resistance to its utilization of the treatment of mastitis [93].

Notably in Ethiopia, the occurrence of methicillin resistance ranges from 24 to 40.9% [94]. In this particular investigation, it was found that the *S. aureus* exhibited the lowest level of resistance towards ampicillin (50%). However, it has been observed that in Japan, the level of resistance towards ampicillin can escalate up to 76.1%∼89.7% [95]. In Nepal, all *S. auerus* isolates demonstrated complete resistance (100%) [96], towards ampicillin, while in Uganda it was recorded to be 73.2% [97] and in India it was found to be 74.42% [98], in India 20.5% [99].

Staphylococcus auerus is recognized for its proficiency in acquiring antimicrobial resistance in both humans and animals. The utilization of beta lactams as the customary antibiotic therapy for *S.aureus* has led to numerous concerns regarding public health. such concerns include the potential for transmission of resistant microorganisms through both the food chain [100,101] and various ecosystems [102]. In addition to posing a threat to public health, the presence of antibiotic residues in milk can prove problematic for the dairy industry, adversely affecting the production technology of fermented milk products [103]. Thus, it is crucial that we increase awareness about the incorporation of a multifaceted One Health approach to address complex issues such as antimicrobial resistance (AMR) to ensure optimal health for humans, animals, and the environment.

This systematic review is subject to several limitations, which must be taken into consideration. Firstly, the review is exclusively focused on a single pathogen, namely *S. aureus*. This limited scope may not provide a comprehensive overview of the broader category of pathogens. Secondly, despite the vast quantity of literature that has been systematically reviewed, the Meta analysis depends on a relatively small sample of articles, with just 14 studies included for the analysis of antimicrobial resistance. This narrow scope may not be representative of the entire body of literature. Thirdly, the meta-analysis conducted was limited to studies in Ethiopia, and a few sample sizes or small numbers of studies were used to estimate the individual beta-lactam antimicrobial resistances. This may limit the generalizability of the findings. It is important to note that our eligibility criteria were limited to articles published in English, which creates potential language bias. Finally, these limitations must be taken into account when assessing the findings of this review.

## 6 Conclusion

This meta-analysis and systematic review estimated the pooled proportion of bovine mastitis associated-*S. aureus* among infection episode in Ethiopia. Hence, this figure revealed that bovine mastitis associated-*S.aureus* is a widely distributed and economically important disease of dairy cows throughout Ethiopia. Subgroup analysis showed that the pooled proportion of bovine associated S.aureus was greater in subclinical mastitis in Northern (Amhara and Tigray region) and central part of Ethiopia in between 2018 and 2022 study years. Furthermore, the overall beta lactamase resistance rate of the bovine associated *S.aureus* was high (68%). Among the beta lactam, the resistance rate of bovine mastitis associated-*S.aureus* was highest in Penicillin. This highest prevalence highest prevalence of *S.aureus* in bovine mastitis and highest resistance rate for the commonly use of beta-lactam antimicrobials should have given big attention. Therefore, this systematic review and meta-analysis was generated concrete and pooled evidence of the distribution of *S. aureus* in bovine mastitis. In general, the summarized findings presented here serve as a valuable tool for healthcare professionals, veterinarians and healthcare organizations alike; enabling them to design and implement appropriate interventions to control and mitigate adverse consequences of *S.aureus* infection in bovine mastitis and to monitor drug resistance *S. aureus* in dairy cows is an essential part of mastitis control.

## Data availability

The datasets generated during and/or analyzed during the current study are available from the corresponding author unreasonable request.

## Authors’ contributions

Melkie Dagnaw Fenta: Data curation, Methodology, Supervision, Formal analysis, Conceptualization, Software, Validation.

Firdyawukal Abuhay Tafere: Conceptualization, writing the original draft and review and editing.

Atsede Solomon Mebratu: Conceptualization, Writing the Original Draft, Methodology.

Birhan Anagaw Malede: Visualization, Formal Analysis, Validation, Supervision.

## Declaration of competing interest

The authors declare that they have no known competing financial interests or personal relationships that could have appeared to influence the work reported in this paper.
