# Supplementary material for: Quarter-wise proportion and beta-lactam resistance rate of bovine mastitis associated- Staphylococcus aureus among infectious episodes in Ethiopia: Systematic review and meta-Analysis
Source: Heliyon. 2023 Jul 17;9(7):e18180. doi: 10.1016/j.heliyon.2023.e18180 (PMC10372662; doi:10.1016/j.heliyon.2023.e18180)
Supplement: Multimedia component 1 [file mmc1.docx]

Supplementary file -1: PRISMA 20202 checklist

| **Section and Topic** | **Item #** | **Checklist item** | **Location where item is reported** |
| --- | --- | --- | --- |
| **TITLE** | | |  |
| Title | 1 | Identify the report as a systematic review. | Page -1 |
| **ABSTRACT** | | |  |
| Abstract | 2 | See the PRISMA 2020 for Abstracts checklist. | checked |
| **INTRODUCTION** | | |  |
| Rationale | 3 | Describe the rationale for the review in the context of existing knowledge. | Page 3-5 |
| Objectives | 4 | Provide an explicit statement of the objective(s) or question(s) the review addresses. | Page- 5, line 108-110 |
| **METHODS** | | |  |
| Eligibility criteria | 5 | Specify the inclusion and exclusion criteria for the review and how studies were grouped for the syntheses. | Page 7 |
| Information sources | 6 | Specify all databases, registers, websites, organisations, reference lists and other sources searched or consulted to identify studies. Specify the date when each source was last searched or consulted. | Page -6, line 130-132 |
| Search strategy | 7 | Present the full search strategies for all databases, registers and websites, including any filters and limits used. | Page 6,139-146 |
| Selection process | 8 | Specify the methods used to decide whether a study met the inclusion criteria of the review, including how many reviewers screened each record and each report retrieved, whether they worked independently, and if applicable, details of automation tools used in the process. | Page 7, line 155-174 |
| Data collection process | 9 | Specify the methods used to collect data from reports, including how many reviewers collected data from each report, whether they worked independently, any processes for obtaining or confirming data from study investigators, and if applicable, details of automation tools used in the process. | Page 8, 194-2020 |
| Data items | 10a | List and define all outcomes for which data were sought. Specify whether all results that were compatible with each outcome domain in each study were sought (e.g. for all measures, time points, analyses), and if not, the methods used to decide which results to collect. | Page 14 line 279-285, figure 1 |
|  | 10b | List and define all other variables for which data were sought (e.g. participant and intervention characteristics, funding sources). Describe any assumptions made about any missing or unclear information. | N/A |
| Study risk of bias assessment | 11 | Specify the methods used to assess risk of bias in the included studies, including details of the tool(s) used, how many reviewers assessed each study and whether they worked independently, and if applicable, details of automation tools used in the process. | N/A |
| Effect measures | 12 | Specify for each outcome the effect measure(s) (e.g. risk ratio, mean difference) used in the synthesis or presentation of results. | N/a |
| Synthesis methods | 13a | Describe the processes used to decide which studies were eligible for each synthesis (e.g. tabulating the study intervention characteristics and comparing against the planned groups for each synthesis (item #5)). | --- |
|  | 13b | Describe any methods required to prepare the data for presentation or synthesis, such as handling of missing summary statistics, or data conversions. |  |
|  | 13c | Describe any methods used to tabulate or visually display results of individual studies and syntheses. | Page 11&12 and table -1 |
|  | 13d | Describe any methods used to synthesize results and provide a rationale for the choice(s). If meta-analysis was performed, describe the model(s), method(s) to identify the presence and extent of statistical heterogeneity, and software package(s) used. | Page 8, 194-2020 |
|  | 13e | Describe any methods used to explore possible causes of heterogeneity among study results (e.g. subgroup analysis, meta-regression). | Page 8, 194-2020 |
|  | 13f | Describe any sensitivity analyses conducted to assess robustness of the synthesized results. |  |
| Reporting bias assessment | 14 | Describe any methods used to assess risk of bias due to missing results in a synthesis (arising from reporting biases). | Page 8, 194-2020 |
| Certainty assessment | 15 | Describe any methods used to assess certainty (or confidence) in the body of evidence for an outcome. | N/A |
| **RESULTS** | | |  |
| Study selection | 16a | Describe the results of the search and selection process, from the number of records identified in the search to the number of studies included in the review, ideally using a flow diagram. | Pagg-9, figure 1 |
|  | 16b | Cite studies that might appear to meet the inclusion criteria, but which were excluded, and explain why they were excluded. | Figure 1 |
| Study characteristics | 17 | Cite each included study and present its characteristics. | Table 1 |
| Risk of bias in studies | 18 | Present assessments of risk of bias for each included study. | Page 14 , figure-3 |
| Results of individual studies | 19 | For all outcomes, present, for each study: (a) summary statistics for each group (where appropriate) and (b) an effect estimate and its precision (e.g. confidence/credible interval), ideally using structured tables or plots. | Page 13 and line 279-285, figure 1 |
| Results of syntheses | 20a | For each synthesis, briefly summarise the characteristics and risk of bias among contributing studies. | Page 21, table 5 |
|  | 20b | Present results of all statistical syntheses conducted. If meta-analysis was done, present for each the summary estimate and its precision (e.g. confidence/credible interval) and measures of statistical heterogeneity. If comparing groups, describe the direction of the effect. | Page 13-21, in the first topic and page -25-28 the results of 2^nd^ topic. With summary of table -7 |
|  | 20c | Present results of all investigations of possible causes of heterogeneity among study results. | Page 15-21 and figure 4-9 |
|  | 20d | Present results of all sensitivity analyses conducted to assess the robustness of the synthesized results. | N/A |
| Reporting biases | 21 | Present assessments of risk of bias due to missing results (arising from reporting biases) for each synthesis assessed. | N/A |
| Certainty of evidence | 22 | Present assessments of certainty (or confidence) in the body of evidence for each outcome assessed. | Page 8 |
| **DISCUSSION** | | |  |
| Discussion | 23a | Provide a general interpretation of the results in the context of other evidence. | Page 29-32 and line 174-278 |
|  | 23b | Discuss any limitations of the evidence included in the review. | N/A |
|  | 23c | Discuss any limitations of the review processes used. | Page 32, lines 275-278 |
|  | 23d | Discuss implications of the results for practice, policy, and future research. | Page 32, lines 272-274 |
| **OTHER INFORMATION** | | |  |
| Registration and protocol | 24a | Provide registration information for the review, including register name and registration number, or state that the review was not registered. | Not registered |
|  | 24b | Indicate where the review protocol can be accessed, or state that a protocol was not prepared. | Not prepared |
|  | 24c | Describe and explain any amendments to information provided at registration or in the protocol. | N/A |
| Support | 25 | Describe sources of financial or non-financial support for the review, and the role of the funders or sponsors in the review. | N/A |
| Competing interests | 26 | Declare any competing interests of review authors. | No competing interest |
| Availability of data, code and other materials | 27 | Report which of the following are publicly available and where they can be found: template data collection forms; data extracted from included studies; data used for all analyses; analytic code; any other materials used in the review. | Available in supplementary files |

**Supplementary file -2: PRISMA 2020 for Abstracts Checklist**

| **Section and Topic** | | **Item #** | Checklist item | **Reported (Yes/No)** |
| --- | --- | --- | --- | --- |
| **TITLE** | | | |  |
| Title | | 1 | Identify the report as a systematic review. | Yes(Page -1) |
| **BACKGROUND** | | | | Yes(Page-2 |
| Objectives | 2 | | Provide an explicit statement of the main objective(s) or question(s) the review addresses. | Yes(Page-2, line 8&9 |
| **METHODS** | With apologies, the selection criteria were not included in the abstract for a multitude of reasons, primarily due to the extensive range of topics covered by the study. Adherence to the journal guidelines has rendered it impracticable to exceed a word limit of 300 in the abstract. | | |  |
| Eligibility criteria | 3 | | Specify the inclusion and exclusion criteria for the review. | No |
| Information sources | 4 | | Specify the information sources (e.g. databases, registers) used to identify studies and the date when each was last searched. | Yes(Page -2  Line 10&11 |
| Risk of bias | 5 | | Specify the methods used to assess risk of bias in the included studies. | Yes(Page-2, line 12 |
| Synthesis of results | 6 | | Specify the methods used to present and synthesise results. | Yes(Page 2, line 12&13) |
| **RESULTS** | **Since the aim of the investigation was twofold, the quantity of studies was distributed across two locations (lines 14 and 21).** | | |  |
| Included studies | 7 | | Give the total number of included studies and participants and summarise relevant characteristics of studies. | Yes(Page 2, line 14) |
| Synthesis of results | 8 | | Present results for main outcomes, preferably indicating the number of included studies and participants for each. If meta-analysis was done, report the summary estimate and confidence/credible interval. If comparing groups, indicate the direction of the effect (i.e. which group is favoured). | Yes(Page-2, line 15-24) |
| **DISCUSSION** | | | |  |
| Limitations of evidence | 9 | | Provide a brief summary of the limitations of the evidence included in the review (e.g. study risk of bias, inconsistency and imprecision). | Yes(Page 2, lines 25-27) |
| Interpretation | 10 | | Provide a general interpretation of the results and important implications. | Yes(Page-2, lines 27&28) |
| **OTHER** | | | |  |
| Funding | 11 | | Specify the primary source of funding for the review. | No |
| Registration | 12 | | Provide the register name and registration number. | No |

Supplementary file-3: AMSTAR-2


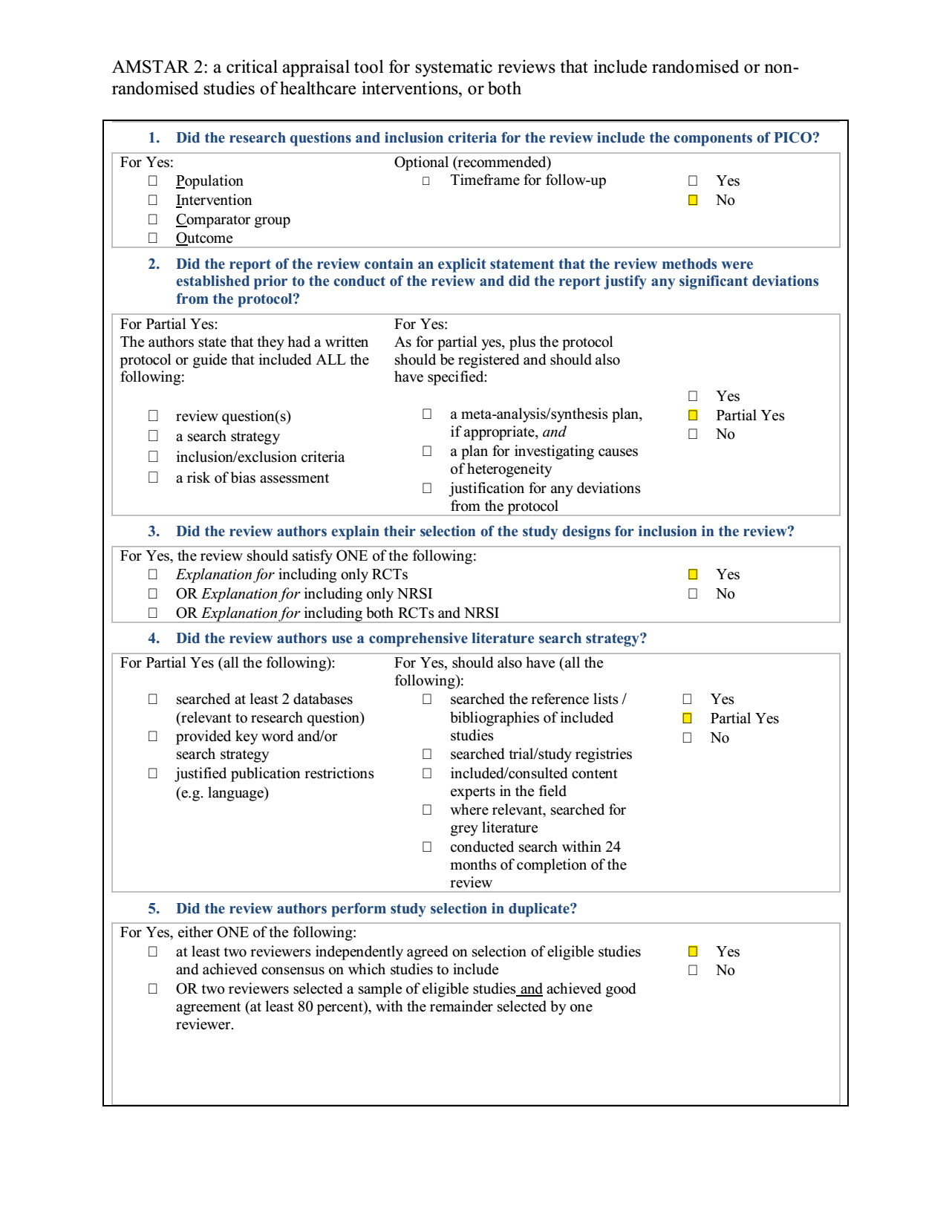


Continued


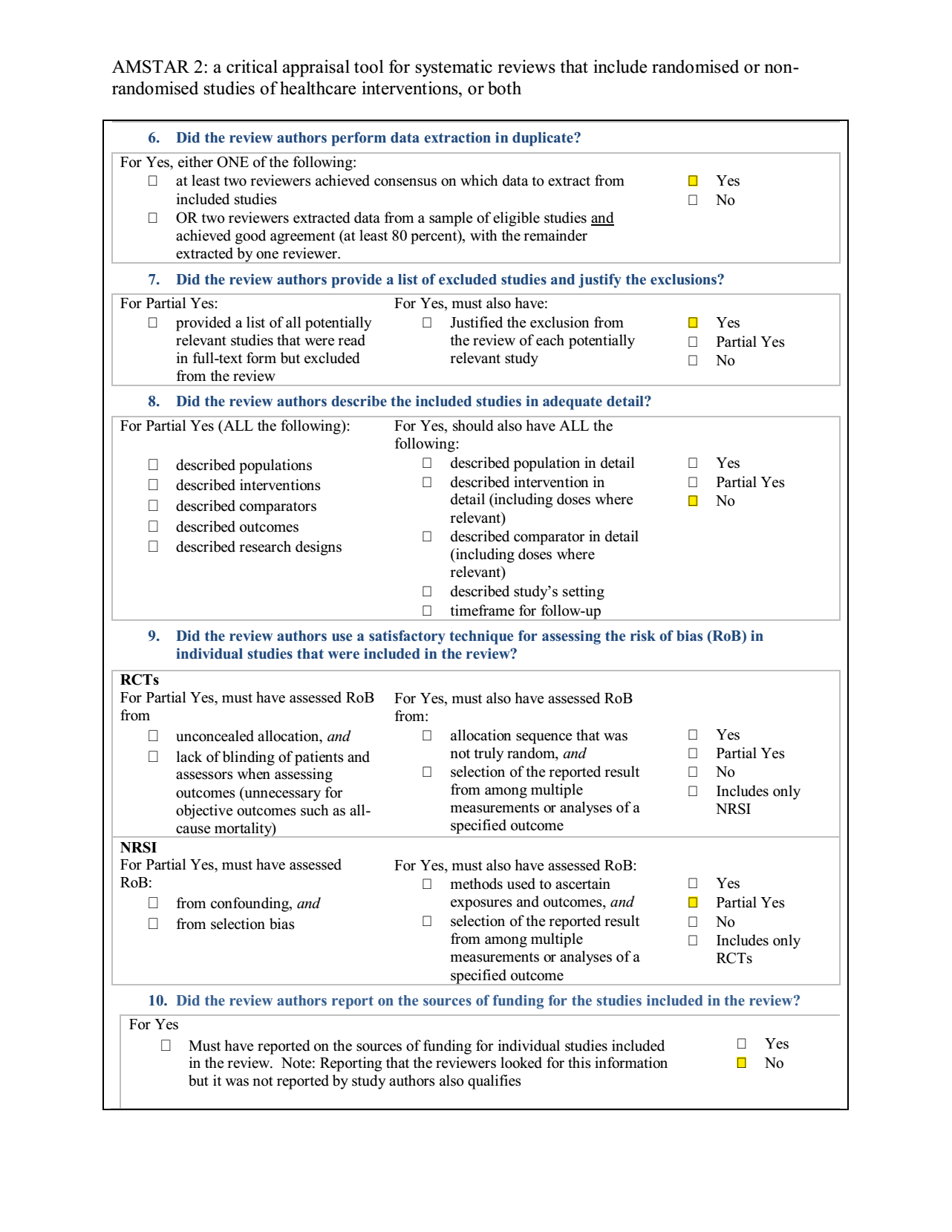


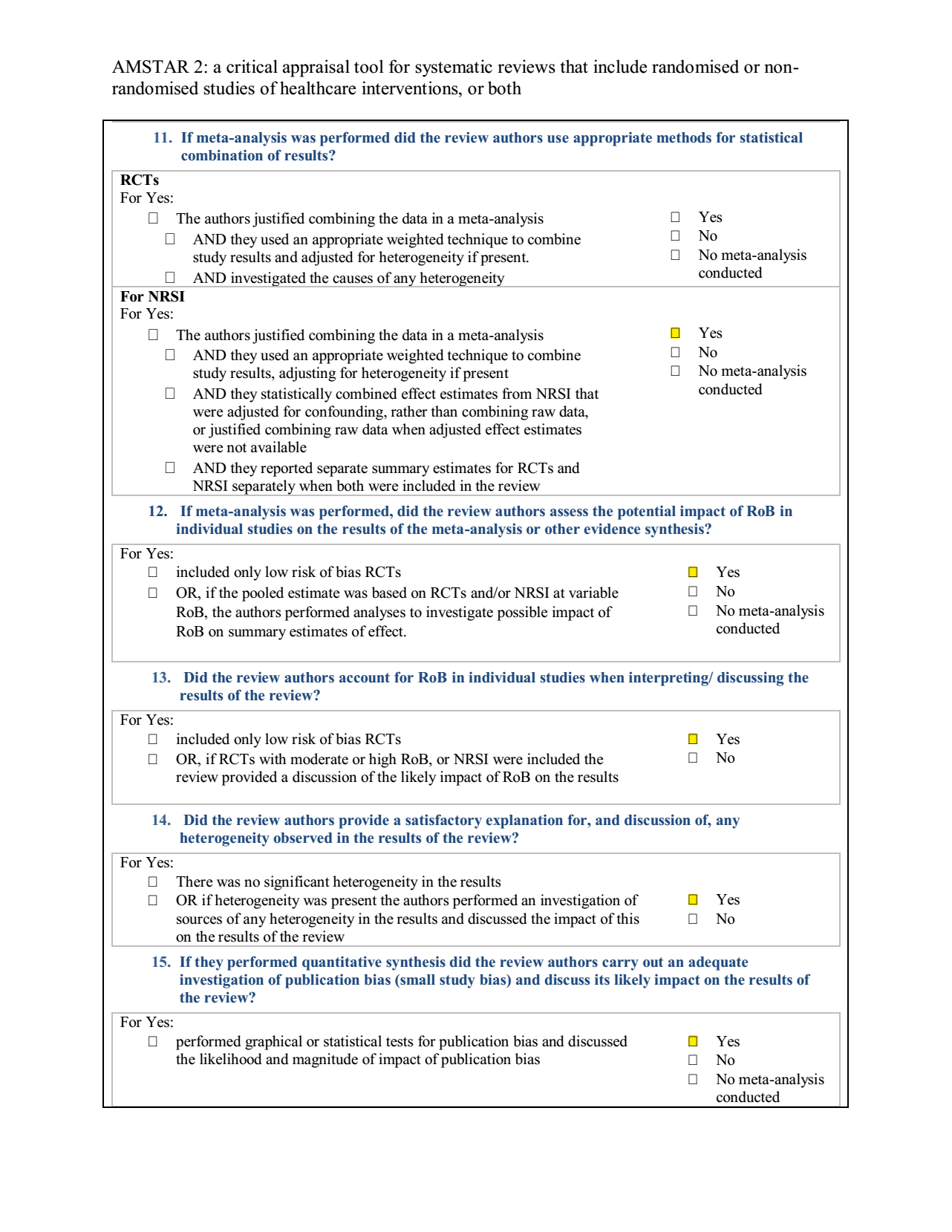


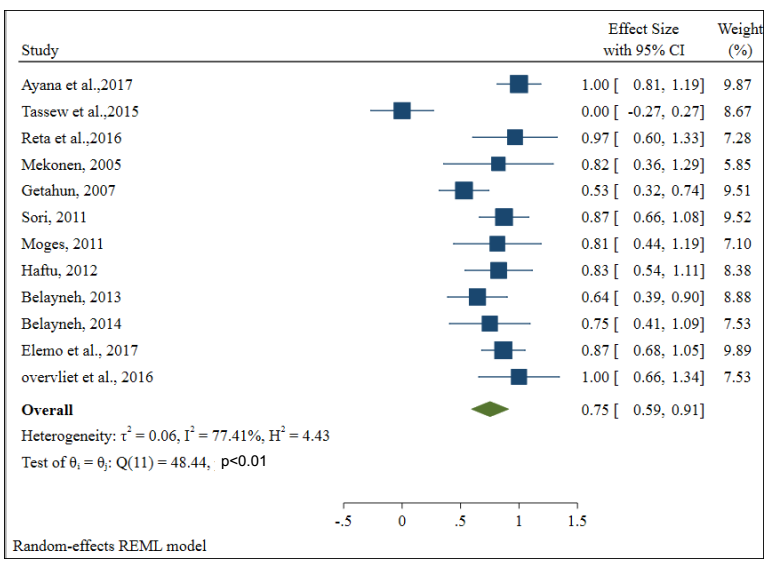


Supplementary Figure 1: forest plot and pooled proportion of *S.aureus* resistance rate for penicillin in the treatment of mastitis in dairy cows


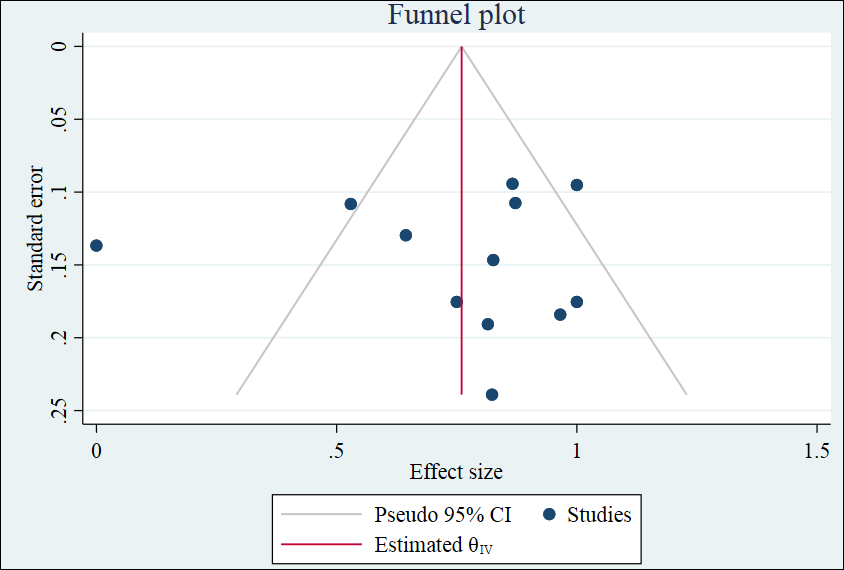


Supplementary Figure 2 Publication bias assessment of penicillin resistance rate of *S.aureus* in the treatment of bovine mastitis


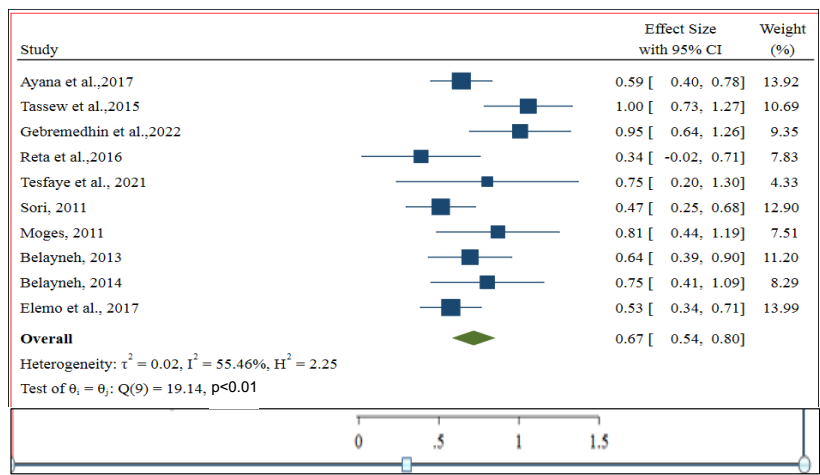


Supplementary Figure 3: forest plot of *S.aureus* resistance to amoxicillin in the treatment of bovine mastitis.


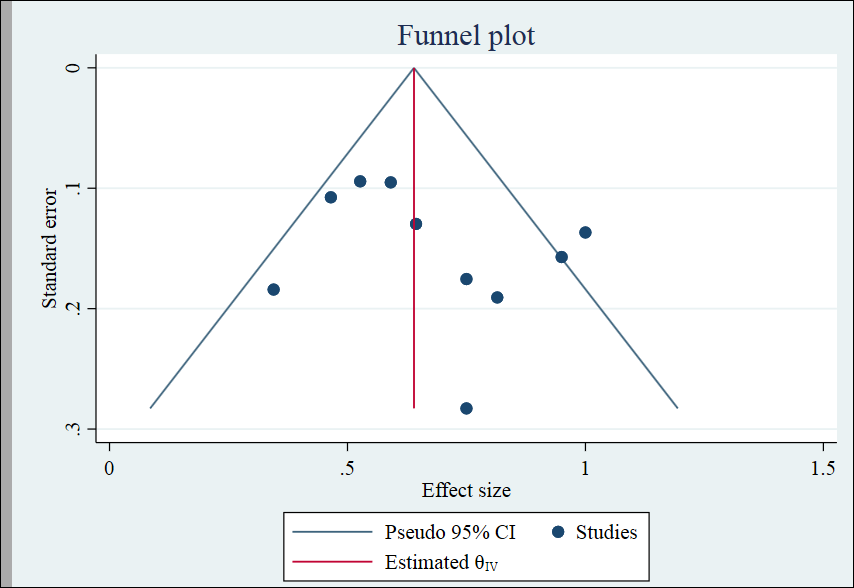


Supplementary Figure 4 funnel plot of amoxicillin resistance rate of S.aureus in the treatment of bovine mastitis


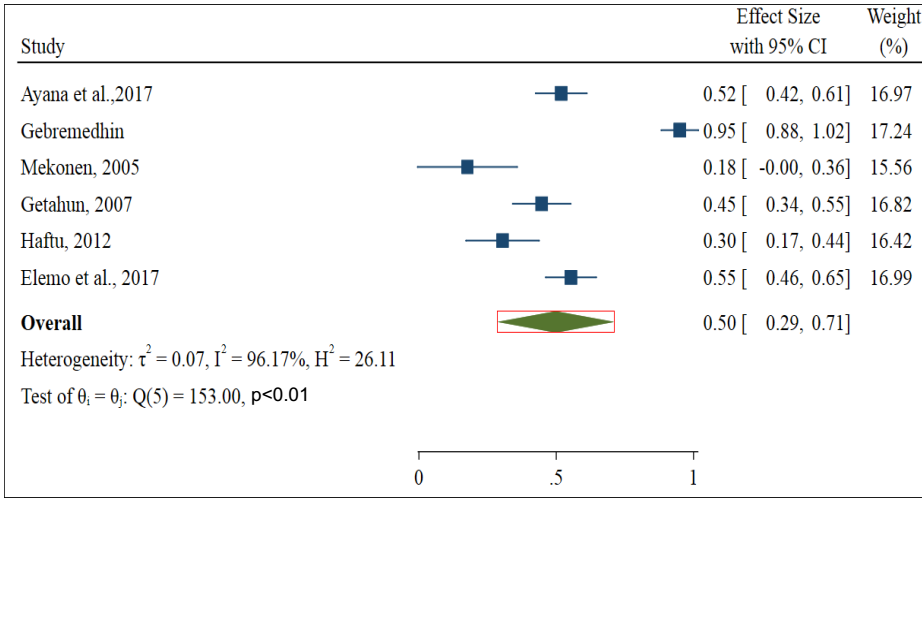
 Supplementary Figure 5: forest plot and pooled proportion of *S.aureus* resistance rate for ampicillin in the treatment of bovine mastitis


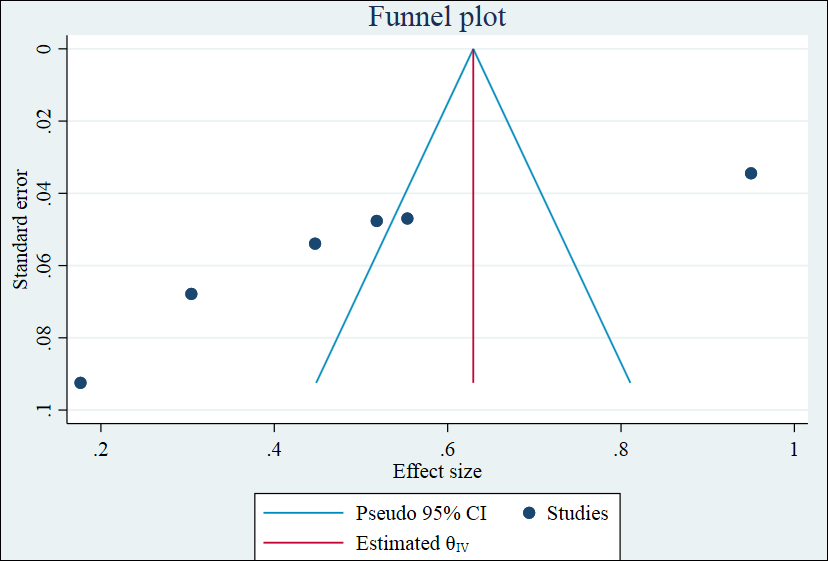


Supplementary Figure 6 funnel plot for bias assessment of ampicillin resistance rate of *S.aureus* in the treatment of bovine mastitis


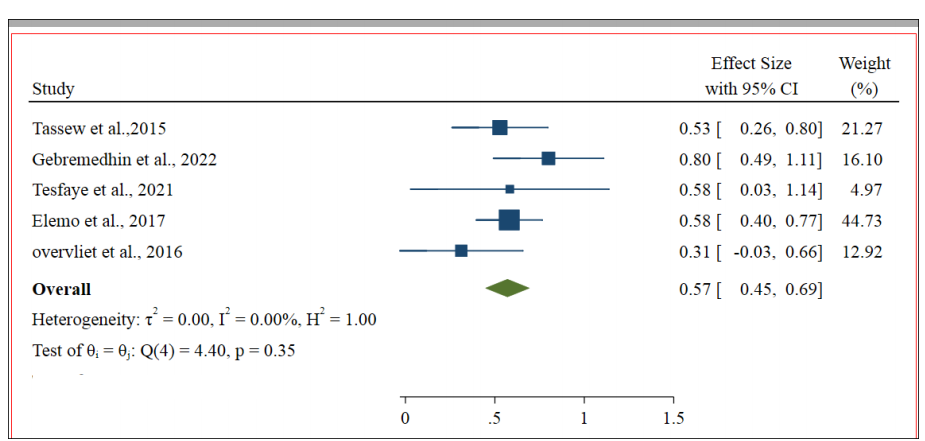


Supplementary Figure 7 forest plot and pooled proportion of *S.aureus* resistance to cephalosporin


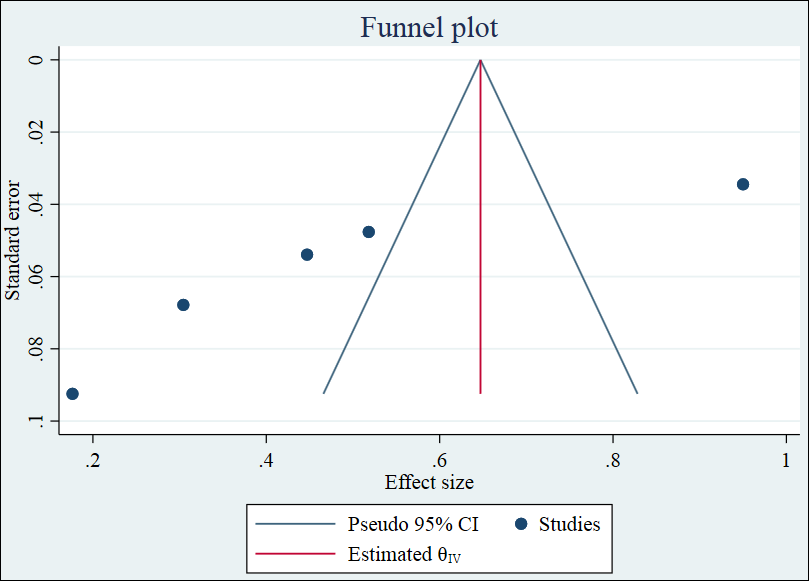


Figure 8 funnel plot of *S.aureus* resistance to cephalosporin
